# Association of Serum Serotonin and Pain in Patients with Chronic Low Back Pain before and after Spinal Surgery

**DOI:** 10.1155/2018/4901242

**Published:** 2018-09-20

**Authors:** Afshin Farhanchi, Behrouz Karkhanei, Negar Amani, Mashhood Aghajanloo, Elham Khanlarzadeh, Zahra Emami

**Affiliations:** ^1^Department of Anesthesiology, Hamadan University of Medical Sciences, Hamadan, Iran; ^2^Department of Neurosurgery, Hamadan University of Medical Sciences, Hamadan, Iran; ^3^Department of Community Medicine, Hamadan University of Medical Sciences, Hamadan, Iran; ^4^Bu-Ali Sina University, Hamadan, Iran

## Abstract

**Introduction:**

In this study we are aiming to evaluate the changes of serum serotonin and its association with pain in patients suffering from chronic low back pain before and after lumbar discectomy surgery.

**Patients and Methods:**

A prospective study was performed on the patients referring to the outpatient clinic in Besat hospital, Hamadan University of Medical Sciences, Hamadan, Iran, during 2016. A 2 mL fasting blood sample was collected from each patient at preoperative day 1 and postoperative day 14 and they were measured for level of serum serotonin. Besides, all patients were asked for severity of their low back pain in preoperative day 1 and postoperative day 14 and scored their pain from zero to ten using a Numerical Rating Scale.

**Results:**

Forty patients with the mean age of 47 ± 13 yrs/old (range 25–77) including 15 (37.5%) males were enrolled into the study. The overall mean score of preoperative pain was significantly decreased from 7.4 ± 2.18 (range 4–10) to the postoperative pain score 3.87 ± 2.92 (range 0–10) (P < .001). The overall levels of pre- and postoperative serum serotonin were 3.37 ± 1.27 (range 1.1–6.4) and 3.58 ± 1.32 (range .94–7.1) ng/mL, respectively, with no significant difference (P = .09). The levels of pre- and postoperative serum serotonin were significantly higher in males and patients older than 50 yrs/old compared to the females and patients younger than 50 yrs/old, respectively (P = .03 and .005, respectively). A significant inverse correlation between the postoperative levels of pain and serum serotonin was observed (r = -.36 and P = .02).

**Conclusion:**

A negative medium strength linear relationship may exist between the postoperative serum serotonin and low back pain.

## 1. Introduction

Low back pain is one the most common health care issues causing enormous financial and social costs globally. Chronic low back pain with duration of pain greater than three months is the second most frequent complaint in physician visits which is related to disability of one-fourth of working populations and significant loss of working days [1, 2]. Lumbar disk herniation is one of the most common causes of chronic low back pain due to mechanical compression of nerve roots and inflammation of intervertebral disks and sometimes surgery is indicated if medical therapy fails to control the disabling symptoms [3, 4].

Although the role of some mechanical, thermal, and chemical agents have been studied in pain regulation, the mechanism of pain induction and perception has not been completely elucidated yet [5]. Recently, serotonin has been proposed to have a role in postoperative pain modulation in both central and peripheral nervous systems. Although postoperative neuropathic pain has been reduced by using selective serotonin reuptake inhibitors (SSRIs) [6], serotonin released from mast cells and platelets due to tissue damage during surgery has been shown to result in pain induction or inhibition [7–9]. To the best of our knowledge, there is no clinical study to find the alterations of serum serotonin in patients suffering from chronic back pain in whom surgical treatment is indicated.

In this study we are aiming to evaluate the effectiveness of lumbar discectomy with either laminectomy or laminotomy surgery on chronic low back pain, changes of pre- and postoperative serum serotonin, and the association of serum serotonin and pain of the patients.

## 2. Patients and Methods

A prospective study was performed on the patients referring to the outpatient clinic in Besat hospital, Hamadan University of Medical Sciences, Hamadan, Iran, during 2016. Inclusion criteria were the patients suffering from chronic low back pain due to lumbar disk herniation who were candidates for surgical treatment. The pain of the patients was disabling even with full medical treatment and was associated with radiculopathy, neurologic deficit, and physical status of class two or less based on the American Society of Anesthesiology classification system. The diagnosis of lumbar disk herniation was made based on the related signs and symptoms of the patients and magnetic resonance imaging findings. Patients with major depression, neurological disorders, diabetes mellitus, and history of using antidepressant or antipsychotic medications were excluded from the study.

A 2 mL fasting blood sample was collected from each patient at preoperative day 1 and postoperative day 14 and was kept at -20°C under the same condition. All samples were measured for level of serum serotonin on the same day using Enzyme-linked immunosorbent assay kit (IBL International Immunoassays GmBH, Germany). Besides, all patients were asked for severity of their low back pain at preoperative day 1 and postoperative day 14 and scored their pain from zero to ten using a Numerical Rating Scale with score of zero indicating having no pain and ten indicating the most severe experienced pain. These data in addition to the demographics and status of smoking and opium use were recorded prospectively. All patients underwent lumbar discectomy with either laminectomy or laminotomy surgery by the same surgeon. Also, all postoperative visits were done by the same surgeon.

The approval of the Ethics Committee of Hamadan University of Medical Sciences was granted prior to patient recruitment and once the aims and procedures of the study were explained, a written informed consent was granted from all participants.

Analysis of data was performed by using the SPSS statistical software (IBM SPSS Statistics for Windows, Version 23.0, IBM Corp., Armonk, NY, USA). Data were expressed as mean ± standard deviation and were compared by using independent or paired samples t-tests. Correlation between the level of serum serotonin and the Numerical Rating Scale score was assessed by using Pearson correlation coefficient. P value less than .05 was considered statistically significant.

## 3. Results

Forty patients with the mean age of 47 ± 13 yrs/old (range 25–77) including 15 (37.5%) males were enrolled into the study. Laminectomy surgery alone has been performed in 18 (45%) patients while laminectomy and lumbar pedicle screw fixation has been performed in the others. Frequencies of the patients who were current cigarette smoker and opium addict were 6 (15%) and 4 (10%), respectively.

The overall mean score of preoperative pain was significantly decreased from 7.4 ± 2.18 (range 4–10) to the postoperative pain score 3.87 ± 2.92 (range 0 – 10) (P < .001). The detailed data about scores of pre- and postoperative pain are shown in Table 1. Moreover, the overall levels of pre- and postoperative serum serotonin were 3.37 ± 1.27 (range 1.1–6.4) and 3.58 ± 1.32 (range .94–7.1) ng/mL, respectively, with no significant difference (P = .09). As shown in Table 2, the levels of postoperative serum serotonin were significantly higher than the related preoperative serum serotonin just in males and patients older than 50 yrs/old, respectively (P = .03 and .005, respectively).

A significant inverse correlation between the postoperative levels of pain and serum serotonin was observed (r = -.36 and P = .02). However, there was no significant correlation between the preoperative levels of pain and serum serotonin (r = .08 and P = .6).

## 4. Discussion

In our study, pain of the patients was significantly reduced from the pre- to the postoperative period regardless of the type of surgery, age, and gender of the patients. Consistent with our data, it has been shown that there is no significant difference between lumbar discectomy surgery with either laminectomy or laminotomy in reducing low back pain [10]. However, the data about relation of age and gender of patients with postoperative outcome is debatable. Like ours, some studies suggested that outcomes of lumbar discectomy in elderly patients were comparable to the younger ones [11, 12]; however, some showed that aging would be associated with adverse outcomes in such patients [10]. Regarding gender, some previous studies, in contrast to ours, suggested that female gender might be associated with poorer outcome [13, 14]. In contrast, a recent randomized and observational study by Kerr et al. showed that gender of the patients was not a predictor of postoperative outcome [15]. Our results also show that the smokers experienced no significant improvement with their low back pain, in contrast to the nonsmokers, which is inconsistent with other studies suggesting better postoperative outcomes for nonsmokers [16, 17].

Serotonin synthesis is controlled by sex hormones; therefore, the alteration of serum serotonin can be expected by difference in ethnicity, gender, and advancement of age [18, 19]. Testosterone and estrogen have been shown to be positively correlated with serum serotonin. The rate of serotonin synthesis in normal males was found to be 52% higher than in normal females. On the other hand, serotonin transporter protein may be reduced in synaptic junctions by the sex hormones and may result in enhancement of synaptic transmission. Thus, the pain perception can be expected to be different between genders and postmenopausal age [19–21]. By advancement of age and consequent decrease in gonadal hormones, serum serotonin regulation by gonadal hormones may be lost and more diverse fluctuation may be seen in stress related conditions. Hence, different levels of sex hormones can be a possible explanation for difference in serotonin levels in different age and gender groups observed in the pre- and postoperative periods of our study. However, further studies including more participants have to be implemented to carefully delineate the observed significant alterations of serum serotonin in different age and gender groups before and after surgery.

In our study, the level of serum serotonin was not significantly changed from the pre- to the postoperative period, in contrast to the level of the pain. Besides, in the postoperative period, the level of serum serotonin was inversely associated with the level of the pain. There is much debate over the association of levels of serum serotonin and chronic low back pain. Similar to ours, an inverse relationship between serum serotonin and pain was reported in healthy individuals and patients performing lumbar exercise for treatment of their low back pain [22, 23]. In an experimental study by Vogel et al., an increment in level of serotonin in a nerve with chronic constriction injury was observed while the level of spinal serotonin decreased [24]. In contrast, some studies showed that the level of spinal serotonin increased or was not changed after spinal nerve injury [25, 26]. Two recent experimental studies have shown that using selective serotonin or serotonin–noradrenaline reuptake inhibitors may be associated with reduction of neuropathic pain related to lumbar disk herniation and inhibition of tumor necrosis factor expression after two or three weeks of treatment suggesting the pronociceptive feature of serotonin in lumbar disk herniation-related pain [27, 28]. On the other hand, in a systematic review by Urquhart et al., in 2008, including nine randomized controlled trials, no significant relief in chronic low back pain was observed in patients who were treated with SSRIs [29]. In contrast, two randomized controlled trials in 2010 suggested that duloxetine as a SSRI associated with improvement of function and pain in patients suffering from chronic low back pain [30, 31].

Chronic low back pain associated with lumbar disk herniation may be related to inflammatory process and mediators such as tumor necrosis factor-alpha and serotonin which are released from mast cells and platelets as a consequence of tissue injury [32]. Serotonin as a neurotransmitter has been proposed to play various roles in pain modulation and signaling mechanisms in both peripheral and central nervous systems. It has been well elucidated that, in peripheral nervous system, serotonin and some mediators of inflammation such as prostaglandins, bradykinin, histamine, potassium ion, and substance P may be responsible for sensitizing the peripheral neurons to inflammation-related pain [33–35]. Nevertheless, in the central nervous system, serotonin may have various effects in stimulating or inhibiting pain signal transmission and its role in nociception in the central nervous system has not been well established yet [36, 37]. The high inconsistency through the various studies may be due to the complex underlying mechanisms of pronociceptive or antinociceptive effects of serotonin in the central nervous system and multiplicity of its receptors. Modulation of tryptophan, precursor of serotonin, has been used as a model for serotonin changes in pain processing [38]. It has been shown that depletion of tryptophan in healthy volunteers diminished morphine induced analgesia or resulted in network disruption similar to disturbance seen in irritable bowel syndrome sufferers with pain [39, 40]. On the other hand, using tryptophan supplementation may be associated with both pain tolerance and mood in healthy subjects [41]. A recent study has indicated an association between reduction of global serotonin and alteration in pain perception, though dissociating these findings from altered mood state [8]. Moreover, it has been reported that patients with chronic tension type headache may have low levels of serum serotonin [42–44]. These data are supported by the fact that using SSRIs may be associated with improvement of chronic pain [45, 46]. Thus, low levels of serotonin may correlate with pain sensitization or perception. However, future studies on the role of serotonin in cognitive and noncognitive characteristics of pain processing and chronic pain is essential to improving individual treatment choice for patients with chronic pain.

## 5. Conclusions

In summary, preoperative chronic low back pain of our patients reduced in postoperative period; however the level of serum serotonin was not significantly changed. Moreover, a negative medium strength linear relationship between the postoperative serum serotonin and pain was observed in our study.

## Figures and Tables

**Table 1:** The detailed data about levels of pre- and postoperative pain based on numerical rating scale.

	**Pain (Numerical Rating Scale score)**		
	**Pre-operation**	**Post-operation**	**P value**
**Surgery**			
** Laminectomy & discectomy**	7.66 ± 2.27	3.72 ± 2.82	**< .001**
** Laminotomy & discectomy**	7.18 ± 2.13	4 ± 3.07	**< .001**
**Gender**			
** Male**	7.66 ± 2.28	4.4 ± 3.45	**.006**
** Female**	7.24 ± 2.14	3.56 ± 2.58	**< .001**
**Age**			
** < 50 yrs/old**	7.21 ± 2.11	3.64 ± 3.12	**.001**
** > 50 yrs/old**	7.5 ± 2.24	4 ± 2.87	**< .001**
**Cigarette smoker**			
** Yes**	7.16 ± 2.63	6.5 ± 3.98	.63
** No**	7.44 ± 2.13	3.41 ± 2.49	**< .001**
**Opium addict**			
** Yes**	7.25 ± 2.36	5.25 ± 4.42	.51
** No**	7.41 ± 2.19	3.72 ± 2.76	**< .001**

Data are shown as mean ± standard deviation.

**Table 2:** The detailed data about levels of pre- and postoperative serum serotonin.

	**Serum serotonin (ng/mL)**		
	**Pre-operation**	**Post-operation**	**P value**
**Surgery**			
** Laminectomy & discectomy**	3.59 ± 1.11	3.62 ± 0.94	.84
** Laminectomy & discectomy**	3.19 ± 1.39	3.56 ± 1.58	.07
**Gender**			
** Male**	3.36 ± 1.05	3.79 ± 1.24	**.03**
** Female**	3.37 ± 1.41	3.46 ± 1.37	.59
**Age**			
** < 50 yrs/old**	3.77 ± 1.18	3.77 ± 1.33	.97
** > 50 yrs/old**	2.62 ± 1.11	3.23 ± 1.27	**.005**
**Current cigarette smoker**			
** Yes**	3.66 ± 0.82	4.17 ± 1.09	.1
** No**	3.32 ± 1.34	3.48 ± 1.34	.25
**Current opium addict**			
** Yes**	3.29 ± 1.17	3.34 ± 1.13	.63
** No**	3.38 ± 1.3	3.61 ± 1.35	.1

Data are shown as mean ± standard deviation.

## Data Availability

According to the regulations of our institution, we are not allowed to share the raw data of our participants publicly.
